# High dimensional biological data retrieval optimization with NoSQL technology

**DOI:** 10.1186/1471-2164-15-S8-S3

**Published:** 2014-11-13

**Authors:** Shicai Wang, Ioannis Pandis, Chao Wu, Sijin He, David Johnson, Ibrahim Emam, Florian Guitton, Yike Guo

**Affiliations:** 1 Discovery Sciences Group, Department of Computing, Huxley Building, Imperial College London, South Kensington Campus, 180 Queen's Gate, London SW7 2AZ, UK

## Abstract

**Background:**

High-throughput transcriptomic data generated by microarray experiments is the most abundant and frequently stored kind of data currently used in translational medicine studies. Although microarray data is supported in data warehouses such as tranSMART, when querying relational databases for hundreds of different patient gene expression records queries are slow due to poor performance. Non-relational data models, such as the key-value model implemented in NoSQL databases, hold promise to be more performant solutions. Our motivation is to improve the performance of the tranSMART data warehouse with a view to supporting Next Generation Sequencing data.

**Results:**

In this paper we introduce a new data model better suited for high-dimensional data storage and querying, optimized for database scalability and performance. We have designed a key-value pair data model to support faster queries over large-scale microarray data and implemented the model using HBase, an implementation of Google's BigTable storage system. An experimental performance comparison was carried out against the traditional relational data model implemented in both MySQL Cluster and MongoDB, using a large publicly available transcriptomic data set taken from NCBI GEO concerning Multiple Myeloma. Our new key-value data model implemented on HBase exhibits an average 5.24-fold increase in high-dimensional biological data query performance compared to the relational model implemented on MySQL Cluster, and an average 6.47-fold increase on query performance on MongoDB.

**Conclusions:**

The performance evaluation found that the new key-value data model, in particular its implementation in HBase, outperforms the relational model currently implemented in tranSMART. We propose that NoSQL technology holds great promise for large-scale data management, in particular for high-dimensional biological data such as that demonstrated in the performance evaluation described in this paper. We aim to use this new data model as a basis for migrating tranSMART's implementation to a more scalable solution for Big Data.

## Background

An ever increasing amount of biological data being produced has predicated the use of databases capable of storing and analyzing such data. In the field of translational research, knowledge management platforms use databases to store a variety of data produced from clinical studies, including patient information, clinical outcomes as well as high-dimensional omics data. For optimal analyzes and meaningful interpretations, such databases also store legacy data taken from public sources, such as the Gene Expression Omnibus (GEO) [1] and the Gene Expression Atlas [2], alongside new study data. This enables cross-study comparisons and cross-validation to take place. High-throughput transcriptomic data generated by microarray experiments is the most abundant and frequently stored data type currently used in translational studies.

Originally developed by Johnson and Johnson for in-house clinical trial and knowledge management needs in translational studies, *tranSMART *is one such knowledge management software platform [3] that has recently been open-sourced. For the needs of various collaborative translational research projects, an instance of tranSMART is hosted at Imperial College London and has been configured to use an Oracle relational database for back-end storage. It currently holds over 70 million gene expression records. When querying the database simultaneously for hundreds of patient gene expression records, a typical exercise in translational studies, the record retrieval time can currently take up to several minutes. These kinds of response times impede analyzes performed by researchers using this deployed configuration of tranSMART. Anticipating the requirement to store and analyze next generation sequencing data, where the volume of data being produced will be in the terabyte (TB) range, the current performance exhibited by tranSMART is unacceptably poor.

A typical query involves searching for and retrieving patient gene/probeset values in a study in order to perform data analysis. For each study, the transcriptomic data stored in the database is comprised of individual probeset values for each patient sample (in some cases multiple samples from each patient are profiled), and annotation information further describing the experiment (e, g. trial information, microarray platform identifier, normalization method, gene descriptions). Once retrieved the data is passed to analytical tools, such as GenePattern [4] or custom R workflows for further analysis or visualization.

Table 1 shows a typical structure of a microarray table that is used in a relational database system. This example is based on the DEAPP schema used in the implementation of tranSMART 1.1. GENE_SYMBOL, PROBESET_ID, PATIENT_ID, TRIAL_NAME correspond to basic annotation data; RAW, LOG and ZSCORE are probeset values [5].

**Table 1:** Relational microarray data schema.

GENE_SYMBOL	PROBESET_ID	PATIENT_ID	TRIAL_NAME	RAW	LOG	ZSCORE
VARCHAR2(100)	VARCHAR2(100)	NUMBER(18)	VARCHAR2(50)	NUMBER(18)	NUMBER(18)	NUMBER(18)

A classic microarray table structure, the DEAPP schema in tranSMART.

All records in the table are organized by a B-tree like structure, a tree data structure that allows search, access, and deletions of tree leaves in logarithmic time. B-tree like structures are commonly used in the implementation of databases and file systems and are fully described in [6]. The time to fetch the patient probeset data using a relational model, such as that in Table 1, is therefore:

$$p_{n}p_{s}\times {\text{log}}_{m}\left(p_{n}p_{s}\right)\times t_{r}$$

where the time to find a next node in the tree is one standard unit, the order of the tree is *m*, the time to fetch a record in relational database is $t_{r}$, number of patients is $p_{n}$, and the number of probesets for each patient is $p_{s}$. For example, if there are 559 patients in a study and each patient has 54,675 probesets, the tree is of order *m*, the total theoretical query time to retrieve all patient probesets, $\sum t_{r}$, is:

$$559\times 54,675\times {\text{log}}_{m}\left(559\times 54,675\right)\times t_{r}$$

What this illustrates is that in order to traverse the tree to retrieve a large study, a significant number of records need to be read from the physical disk where such a large number of database operations will cumulatively be slow. A common solution for scalability issues in relational databases is database partitioning [7], which allows for a logical database to be divided into constituent parts and distributed over a number of nodes, for example in a compute cluster. This approach has enabled relational databases to support large-scale genomics datasets through horizontal partitioning (also referred to as *sharding*), however this still does not solve the data retrieval performance issues such as those observed in translational research studies loaded into tranSMART.

An alternative solution, and what forms the contribution described in this paper, is to use a non-relational database modality, the key-value pair data model, as implemented in NoSQL databases such as Google BigTable [8], a column-oriented storage system for structured data. BigTable uses a Row Key and a Column Key to locate a Value. The first advantage of such a data model is that it typically maintains data in lexicographic order by Row Key. The second advantage is that the Column Key includes two parts: a Family and a Qualifier, where database columns (Qualifiers) are grouped into sets (Families) and all data stored in a Family is usually of the same type and is compressed and stored together. When a key is retrieved, a key-value array, termed a StoreFile, is loaded into memory and the expected key-value pairs are returned. Taking these features of the key-value model into account, we hypothesized that a key-value pair data model would more performant in data retrieval than the current relational model used for microarray data storage in tranSMART. In this paper we describe an experiment using a new database model for one of tranSMART's microarray data tables that may be more suitable for high-dimensional data storage and querying than the relational model currently implemented in tranSMART.

## Related work

The current version of tranSMART operates with SQL-based databases in a single node mode, typically as single PostgreSQL [9] 9.3 or Oracle 11g [10] database. It is possible to migrate the current tranSMART data model to SQL-based database clusters to solve the performance problem mentioned in motivating example. For example, MySQL Cluster [11] is an open-source high performance database cluster. MySQL Cluster consists of multiple network database nodes (NDB), a single management node (MGM) and multiple MySQL nodes. The data is horizontally partitioned across the NDBs, where each MySQL node is responsible for handling SQL operations. The MGM controls all NDB and MySQL nodes. MySQL Cluster can use the same relational data model detailed previously in Table 1.

**Figure 1:**
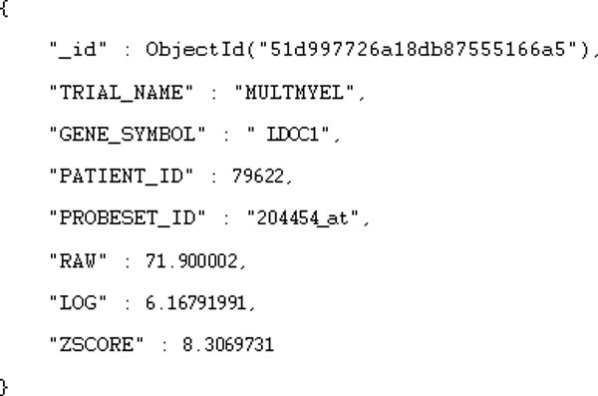
**JSON example**. Example of a JSON object that maps to the patient record illustrated in Table 2.

NoSQL document storage systems, such as MongoDB [12], CouchDB [13], Riak [14] are popular alternatives to using relational database management systems such as MySQL, because relational data models can easily map to a document-based key-value model. For example, MongoDB has features such as indexing in the form of B-trees, auto-sharding and asynchronous replication of data between servers. MongoDB stores data in collections and each collection contains documents. Each document is a serialized JSON [15] object. Our MongoDB implementation is similar to the relational model where each column is represented with a field in a JSON object. For example, the relational data record in Table 2 maps to the JSON object shown in Figure 1. Note that the field _id holds a unique key generated for each JSON object.

**Table 2:** Example of a relational model representation of a patient record.

GENE_SYMBOL	PROBESET_ID	PATIENT_ID	TRIAL_NAME	RAW	LOG	ZSCORE
LDOC1	204454_at	79622	MULTMYEL	71.900002	6.16791991	8.3069731

All of the data is imported into a single collection with the index built on the keys, where the sharding key uses PATIENT_ID to distribute the data into multiple ranges based on PATIENT_ID.

NoSQL systems for structured data, such as BigTable and Cassandra [16], have been developed for managing very large amounts of data spread out across many servers. In contrast to document key-value storage, such as MongoDB, NoSQL structured data systems do not easily map from a relational data model to a key-value model. HBase [17] is a popular example of such a system where it is part of the Hadoop framework that has been widely adopted for large-scale storage and processing. HBase is an Apache Software Foundation open source project written in the Java programming language, and provides BigTable-like capabilities. There are three major components of HBase. The HBase Master assigns Regions to HRegion Servers. HRegion Servers handle client read and write requests. The HBase client discovers HRegionServers, which are serving a particular row range of interest. Our microarray data model implementation using HBase is described in our Results section.

## Results

We designed a key-value [18] based data model to support faster queries over large-scale microarray data, and implemented the schema using HBase. We compared the query retrieval time against a traditional relational data model running on a MySQL Cluster database, and a relational model running on a MongoDB key-value document database.

### Schema design

#### Determining Row Key and Column Key of transcriptomic data

In order to speed up queries, the TRIAL_NAME and PATIENT_ID are placed in the Row Key. This is for two reasons. The first reason is that a Family can manage a single data type (RAW, LOG, or ZSCORE) of data efficiently. When a Family is used in this way, all attributes larger than the data type (TRIAL_NAME and PATIENT_ID) are placed in the Row Key and attributes smaller than the data type (GENE_SYMBOL and PROBESET_ID) are placed in the Qualifier. The second reason is that all records belonging to a patient are stored together in several StoreFiles. For a typical use case when multiple identical PATIENT_IDs are retrieved, the StoreFiles storing these patients' data will be loaded into BigTable caches. Therefore, only retrieving the first record causes a significant delay when loading data from disk to memory, while all other records in the same StoreFile are fetched directly from memory caches. This design takes full advantage of BigTable caches.

#### Optimizing the Row Key to speed up data location

The order of the two fields in the Row Key also has a very significant effect on the query performance. Typical user cases always begin with a gene information query of several patients. By placing the TRIAL_NAME before the PATIENT_ID (TRIAL_NAME + PATIENT_ID), the composite key becomes two indices on the trial and patient respectively. When multiple identical patients are retrieved, TRIAL_NAME + PATIENT_ID can precisely locate all StoreFiles expected by using TRIAL_NAME + PATIENT_ID to compare to the Start and End keys of a StoreFile.

#### Optimizing the Column Key to increase cache hit rate

One way to further increase cache hit rate is to design a new structure by classifying different types of data under Column Key. The Family divides different types of data (RAW, LOG, and ZSCORE) into different StoreFiles and the Qualifier (GENE_SYMBOL + PROBESET_ID) orders probeset values lexicographically. In the motivating example above, the user requests data on several patients that contains only one type of probeset, but the query returns millions of records. Such large numbers of probeset records can be loaded with only a limited number of disk loading operations through the corresponding Column Keys.

#### Key-value data model example

To better explain our design, we can illustrate its application using the relational example in Table 3 and transforming it into our key-value model shown in Table 4. Table 3 shows how a StoreFile stores these key-value data on physical disks. Key-value pairs are ordered alphabetically by the Key in Table 3 and each pair is an array of Java data type byte.

**Table 3:** Example in DEAPP

GENE_SYMBOL	PROBESET_ID	PATIENT_ID	TRIAL_NAME	RAW	LOG	ZSCORE
LDOC1	204454_at	79622	MULTMYEL	71.900002	6.16791991	8.3069731

EI24	216396_s_at	79622	MULTMYEL	917.20001	9.84109256	9.57629541

GRID1	1555267_at	79737	MULTMYEL	608.29999	9.24863917	8.88386512

**Table 4:** Example data model in BigTable transformed from table 3.

Key			Value
**Row Key**	**Column Key**		
		
	**Family**	**Qualifier**	

MULTMYEL + 79622	LOG	EI24 + 216396_s_at	9.84109256

MULTMYEL + 79622	LOG	LDOC1 + 204454_at	6.16791991

MULTMYEL + 79737	LOG	GRID1 + 1555267_at	9.24863917

4a. LOG Family StoreFile			

**Key**			**Value**

**Row Key**	**Column Key**		
		
	**Family**	**Qualifier**	

MULTMYEL + 79622	RAW	EI24 + 216396_s_at	917.20001

MULTMYEL + 79622	RAW	LDOC1 + 204454_at	71.900002

MULTMYEL + 79737	RAW	GRID1 + 1555267_at	608.29999

4b. RAW Family StoreFile			

**Key**			**Value**

**Row Key**	**Column Key**		
		
	**Family**	**Qualifier**	

MULTMYEL + 79622	ZSCORE	EI24 + 216396_s_at	9.57629541

MULTMYEL + 79622	ZSCORE	LDOC1 + 204454_at	8.306973

MULTMYEL + 79737	ZSCORE	GRID1 + 1555267_at	8.88386512

4c. ZSCORE Family StoreFile			

For example, if the raw intensity values of all patients in the trial MULTMYEL are retrieved, the key-value system will locate the Key,

Trial Name (MULTMYEL) + minimum Patient ID(00000) + Family (Raw) + Gene(00000000) + Probeset(00000000)

and then load a StoreFile containing Key1

MULTMYEL + 79622 + 10001 + RAW + EI24 + 216396_s_at into memory. This StoreFile probably also contains Key2 consisting of

MULTMYEL + 79622 + 10001 + RAW + LDOC1 + 204454_at because Key2 is typically stored adjacent to Key1 in the physical storage. This results in a reduction in query response time by increasing the cache hit rate.

As in the relational model, key-value data is organized as a B-tree. Each leaf in the tree is a StoreFile (128 MB by default), each of which contains data from more than one patient. Considering the maximum probeset count in our database of 54,675 probesets per sample using the GPL570 platform, where the average probeset data size is 300 bytes, we can see that,

300B×54,675≈16MB<128MB

In this case, one type of probeset values for a patient can at most be stored in two StoreFiles. Therefore we assume each patient data in one type is stored within two StoreFiles. The time to fetch patients data is less than:

$$2p_{n}\times {\text{log}}_{m}\left(p_{n}\right)\times t_{kv}$$

where the time to find a next node in the tree is one standard unit, the tree is of order *m*, the time to fetch a StoreFile in a key-value system $t_{kv}$, and patient number is $p_{n}$. Thus, for example, if there are 559 patients in a study and each patient has 54,675 probesets, the total theoretical query time $\sum t_{kv}$ is:

$$2\times 559\times {\text{log}}_{m}\left(559\right)\times t_{kv}$$

In order to make a full comparison of time consumption between key-value data model and relational data model, we assume $t_{r}$ is the time to load 32 KB (MySQL Cluster's data page size) data from physical disk into memory and $t_{kv}$ is the time to load 128 MB data, then in a common physical server with a SATA disk (X79 series chipset 6-Port SATA AHCI Controller), $t_{r}$ is approximately 10 milliseconds and $t_{kv}$ is approximately 9,000 milliseconds. In this case:

$$\frac{\sum t_{r}}{\sum t_{kv}}\approx \frac{82.76}{1}$$

Therefore, if cache and query optimizers are not taken into account, we estimate theoretically that an ideal key-value data model may be up to about 83 times faster than an ideal relational data model. The actual observed speedup is much less than our theoretical estimate since real-world performance will be vary according to implementation. However as the basis of our hypothesis, based on our theoretical calculations we propose that a key-value will be significantly faster than a relational model when querying microarray data such as that stored in tranSMART.

### Querying transcriptomic data in tranSMART

Our experiment was performed using a dataset loaded in an instance of tranSMART running on a cloud computing test-bed, IC Cloud [19], installed at Imperial College London. In our experiment we took a database dump of the DEAPP schema that tranSMART uses to store patient microarray data and extracted a public Multiple Myeloma (MULTMYEL) [20,21] dataset [GEO:GSE24080] [22]. The reason we chose MULTMYEL as our test dataset was that it is one of the largest datasets loaded into our tranSMART database instance, consisting of 559 samples and 54,675 probesets for each sample, totalling approximately 30.5 million records. We took this dataset and transformed and loaded it into two different key-value databases, HBase and MongoDB, and a relational database, MySQL Cluster, each of which was running on IC Cloud. HBase was used to implement our key-value model, while MongoDB and MySQL Cluster both re-implement the relational model. Each database was configured as follows:

• **HBase (Version 0.96.0 on Hadoop 1.0.3)**: One master server node and three slave nodes with HBase configured in *fully distributed *mode. The master server was configured as a virtual machine (VM) with 4 CPU cores and 8 GB memory, while each slave node was configured as VMs with 2 CPU cores and 4 GB memory. Each VM used a 100 GB disk. Three copies is the minimum number for the Hadoop Distributed File System to guarantee data consistency. We ran queries in HBase using two read methods, Random Read and Scan, in order to select the faster method. In the Random Read method each patient's data is retrieved discretely. In Scan, all patients from the first Row Key to the last Row Key expected are retrieved sequentially, including unexpected Row Keys between two expected ones. However we found Scans over sequential records are significantly faster than Random Reads.

• **MongoDB (Version 2.2.4)**: Four VMs were used in our MongoDB cluster (VM A configured with 4 CPU cores and 8 GB memory, VMs B, C and D with 2 CPU cores and 4 GB memory). Each VM used a 100 GB disk. VM A and B formed a replication set (rs0), with C and D forming another (rs1); rs0 and rs1 formed a two-shard sharding cluster. VMs B, C and D were also deployed as three configuration servers (mongod --configsvr). VM A hosted the load balancer (mongos), and handled all the data operation requests. MongoDB requires two copies to guarantee data consistency. MongoDB indexes the TRAIL_NAME and PATIENT_ID fields within JSON objects.

• **MySQL Cluster (Version 5.6.11-ndb-7.3.2)**: Four VMs, with one as a manager node and three data nodes. The manager node consists of a MGM, a MySQL and a NDB using a VM with 4 CPU cores and 8 GB memory. Each VM used a 100 GB disk. Each data node consisted of a NDB. Disk-based InnoDB storage engine was used, where indexed columns and indices are stored in memory and non-indexed columns are stored in disks. The TRIAL_NAME and PATIENT_ID were indexed by BTREE to speed up the query operations. Two data copies were used to guarantee its data consistency.

### Gene data query using a large transcriptomic dataset

**Figure 2:**
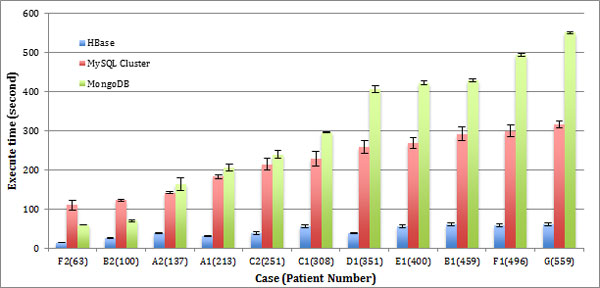
**Performance of key-value vs. relational data model**. The bar chart shows the query retrieval times for each of the test cases over varying numbers of patient record queries. The NoSQL model implementation on HBase performs the best with an approximately 3.06~7.42-fold increase in query performance than relational model on MySQL Cluster and 2.68~10.50-fold increase than the relational model on MongoDB.

In order to assess HBase, MongoDB and MySQL Cluster performances using a gradient of retrieval size requests, we devised a series of typical marker selection queries based on relevant biological questions [23] and data download requests:

• **Test cases A1 and A2: **213 patients who underwent Therapy 2 and survived longer than 30 months are compared to 137 patients who undertook the same therapy and survived less than 30 months, to discover gene expression patterns affecting the short term response to therapy 2.

• **Test cases B1 and B2: **459 patients who took Therapy 2 and were still alive at the end of the trial were compared to 100 patients who took Therapy 2 and did not survive the duration of the study, to discover gene expression patterns affecting the long term response to therapy 2.

• **Test cases C1 and C2: **308 patients who lived longer than 26 months were compared to 251 patients who survived less than 26 months, to discover gene expression patterns affecting patient survival.

• **Test case D1 and D2: **351 patients who took Therapy 2 were compared 208 patients who took Therapy 3. This comparison is used to see the different effect of the two therapies. D2 is comparable to test case A1, as it retrieves a similar amount of patients and thus is not shown on Figure 2.

• **Test case E1 and E2: **400 patients who survived less than 36 months were compared to 159 patients who survived more than 36 months. E2 is comparable to test case A2, as it retrieves a similar amount of patients and thus is not shown on Figure 2.

• **Test case F1 and F2: **496 patients who lived less than 52 months were compared to 63 patients who survived beyond 52 months.

• **Test case G: **A query over the whole clinical trial consisting of all 559 patients. This last case is used to download all patient information to perform further analysis in external tools.

We tested each case three times, where after every test we shut down the entire cluster to clean all caches to remove the influence of residual cached data. The record retrieval times for the HBase, MySQL Cluster and MongoDB configuration are shown in Figure 2. In this experiment only RAW data was targeted.

With the number of records increasing, most test cases in relational model show retrieval times also increasing.

MySQL Cluster retrieval times are slow when querying fewer numbers of patient records with query times increasing slightly as the data queries scale up. Our key-value based data model demonstrates an average 5.24 times of increase compared to the relational model implemented on MySQL Cluster. In 6/11 of cases, the key-value data model is more than 5x faster than relational model on MySQL Cluster. In 10/11 of cases, the key-value data model is more than 4x faster than the relational model on MySQL Cluster. In the worst case, A2, the key-value data model is more than 3.61 times faster than relational model on MySQL Cluster. As the size of the queried data scales upwards, MySQL Cluster performs less stable than other databases as show by the greater average result deviations in Figure 2.

The relational model on MongoDB performs well with smaller queries but slows significantly when the amount of data retrieved is scaled up. Our key-value based data model demonstrates an average 6.47 times increase in query performance compared to the relational model implemented on MongoDB. In 6/11 of cases, key-value retrieval is more than 6x faster than that on MongoDB, especially in cases with large retrieval data. In 10/11 of cases, the key-value retrieval speed is more than 4x faster than that on MongoDB. In the worst case, B2, the key-value data model is more than 2.68 times faster than the relational model on MongoDB.

**Figure 3:**
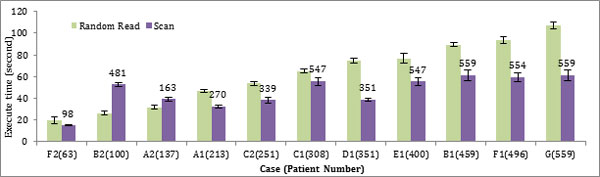
**Performance of Random Read vs. Scan in key-value data model**. The bar chart shows the query retrieval times for each of the test cases over varying numbers of patient record queries using both Random Read and Scan methods. The numbers above scan bar show the patient numbers the scan read in that case. The error bar shows the deviation of each test.

The data retrieval time in the key-value model varies more widely due to the different read operations, Random Read and Scan, and also the Scan range in different cases, as shown in Figure 3. In most test cases in our experiment, Scans are quicker than Random Reads. For Scans, the worst case is when the expected patient count is small and the patient record distribution within the database is very sparse. In this situation, the Scan operation degrades to sequentially read all patient data. For example, in case B2, 63 patient records are expected and distributed over a range of 481 patients. However, a whole dataset Scan only needs about half of the time to perform a Random Read.

**Figure 4:**
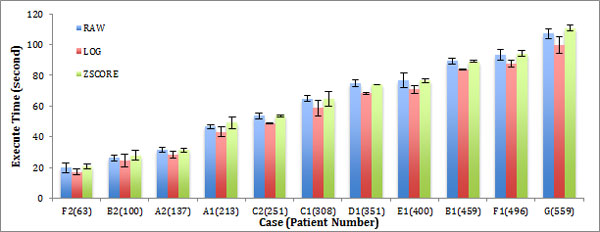
**Performance of Random Read in different Families**. The bar chart shows the query retrieval times for each of the test cases over varying numbers of patient record queries of three Families. The error bar shows the deviation of each test.

**Figure 5:**
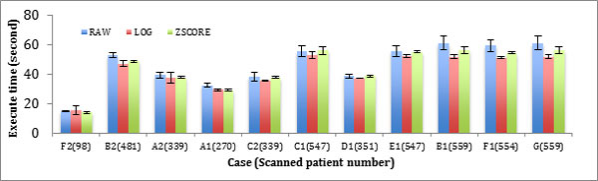
**Performance of Scan in different Families**. The bar chart shows the query retrieval times for each of the test cases over varying numbers of patient record queries of three Families. The error bar shows the deviation of each test.

The situation is similar when retrieving other types of data, such as LOG or ZSCORE, as shown in Figure 4 and 5. In these two figures, the performance trend observed for each Family is similar. Only the deviation values vary. Thus, compared to the other databases, the same conclusion will be generated for every Family.

## Discussion

Our results show that in general our key-value implementation of the tranSMART DEAPP schema using HBase outperforms the relational model on both MySQL Cluster and MongoDB, as we originally hypothesized. HBase's increased performance in comparison to MySQL Cluster comes from the new key-value data model. This new key-value data model is however currently not efficient enough for discrete data queries. A possible solution is the use of the HBase feature, CCIndex [24]. Discrete query operations are generated by WHERE conditions in SQL queries, where CCIndex can index columns that are frequently used in those conditions and transform these discrete queries into large range scans. As future work, we plan to integrate CCIndex to further improve the performance in our key-value model for high-dimensional data in tranSMART.

Queries in HBase are optimized manually by choosing to perform either a pure Scan or pure Random Read operation. Ideally there should be a query optimizer built into the HBase system, like in MySQL Cluster or MongoDB, to automatically generate query plans for high-dimensional data retrieval. Pure Scans or Random Reads may not be the best choice for certain cases, where a mixed, dynamically selected query method may perform better than statically choosing one or the other.

Our results also show that performance in different Families differ slightly. Family LOG performs best in almost all cases. This phenomenon may result from the LOG StoreFile location in HBase system.

We also observed that the relational model implementation consumes a lot of memory. Although MySQL Cluster supports disk based storage, all indexed columns and indices are still stored in memory. This feature may influence its scalability.

## Conclusions

Our work is aimed at solving the performance issues faced in translational research data storage databases, due to the increasing volume of data being produced. In this paper we have demonstrated that a key-value pair schema leads to an increase in performance when querying high-dimensional biological data, over the relational model currently implemented in tranSMART. Our results show that, in general, our key-value implementation of the tranSMART DEAPP schema using HBase outperforms the relational model on both MySQL Cluster and MongoDB. We aim to further optimise the schema design to achieve a better performance and use this schema as the prototype for developing a next generation sequencing storage data model for the tranSMART data warehouse.

## Competing interests

The authors declare that they have no competing interests.

## Authors' contributions

SW designed the key-value data model, ran the HBase experiment, analyzed the HBase results and drafted the manuscript. IP helped to define the research theme, designed and explained the test cases, and drafted the manuscript. CW designed the MongoDB data model, ran the MongoDB experiment and analyzed the MongoDB results. SH designed the MySQL Cluster data model, ran the MySQL Cluster experiment and analyzed the MySQL Cluster results. DJ participated in the key-value data models design and drafted the manuscript. IE participated in the key-value data model design and the test case design. FG participated in MongoDB data model design and experiment. YG defined the research theme and participated in all the models design. All authors read and approved the final manuscript.

## Declarations

This research was partially supported by the Innovative R&D Team Support Program of Guangdong Province, China (No. 201001D0104726115) and the eTRIKS consortium (*Delivering European Translational research Information & Knowledge Management Services*, Grant Agreement No. IMI 115446). SW is also supported by a scholarship awarded by the China Scholarship Council from 2012-2014. The funding agencies had no role in the design, collection, analysis or interpretation of the data, in preparation of the manuscript, or in the decision to submit the manuscript for publication.

This article has been published as part of *BMC Genomics *Volume 15 Supplement 8, 2014: Selected articles from the 9th International Symposium on Bioinformatics Research and Applications (ISBRA'13): Genomics. The full contents of the supplement are available online at http://www.biomedcentral.com/bmcgenomics/supplements/15/S8.

## References

[B1] Barrett T Wilhite SE Ledoux P Evangelista C Kim IF Tomashevsky M Marshall K a Phillippy KH Sherman PM Holko M Yefanov A Lee H Zhang N Robertson CL Serova N Davis S Soboleva A NCBI GEO: archive for functional genomics data sets--update Nucleic Acids Res 2013 41 Database D991 5 23193258 10.1093/nar/gks1193 PMC3531084

[B2] Kapushesky M Adamusiak T Burdett T Culhane A Farne A Filippov A Holloway E Klebanov A Kryvych N Kurbatova N Kurnosov P Malone J Melnichuk O Petryszak R Pultsin N Rustici G Tikhonov A Travillian RS Williams E Zorin A Parkinson H Brazma A Gene Expression Atlas update -- a value-added database of microarray and sequencing-based functional genomics experiments Gene Expr 2012 40 1 5 10.1093/nar/gkr913 PMC3245177 22064864

[B3] Szalma S Koka V Khasanova T Perakslis ED Effective knowledge management in translational medicine J Transl Med 2010 8 68 10.1186/1479-5876-8-68 20642836 PMC2914663

[B4] Reich M Liefeld T Gould J Lerner J Tamayo P Mesirov JP GenePattern 2.0 Nat Genet 2006 500 501 16642009 10.1038/ng0506-500

[B5] Cheadle C Vawter MP Freed WJ Becker KG Analysis of microarray data using Z score transformation J Mol Diagn 2003 5 73 81 10.1016/S1525-1578(10)60455-2 12707371 PMC1907322

[B6] Bayer R McCreight EM Organization and maintenance of large ordered indexes Acta Inform 1972 173 189

[B7] Eadon G Chong EI Shankar S Raghavan A Srinivasan J Das S Supporting table partitioning by reference in Oracle Proc ACM SIGMOD Int Conf Manag Data 2008 1111 1122

[B8] Chang F Dean J Ghemawat S Hsieh WC Wallach DA Burrows M Chandra T Fikes A Gruber RE Bigtable: A Distributed Storage System for Structured Data ACM Trans Comput Syst 2008 26 1 26

[B9] Momjian B PostgreSQL: Introduction and Concepts 2001 22 462

[B10] Oracle http://www.oracle.com/index.html

[B11] MySQL Cluster CGE http://www.mysql.com/products/cluster/

[B12] Dede E Govindaraju M Gunter D Performance evaluation of a MongoDB and hadoop platform for scientific data analysis Proc 4th ACM Work Sci cloud Comput 2013 13

[B13] Anderson JC Lehnardt J Slater N CouchDB: The Definitive Guide 2010 5 272

[B14] Riak http://docs.basho.com/

[B15] JSON http://www.json.org/

[B16] Lakshman A Malik P Cassandra: a decentralized structured storage system ACM SIGOPS Oper Syst Rev 2010 44 35

[B17] George L HBase The Definitive Guide 2008

[B18] Stonebraker M SQL databases v. NoSQL databases Commun ACM 2010 10

[B19] Guo LGL Guo YGY Tian XTX IC Cloud: A Design Space for Composable Cloud Computing Cloud Comput (CLOUD), 2010 IEEE 3rd Int Conf 2010

[B20] Raab MS Podar K Breitkreutz I Richardson PG Anderson KC Multiple myeloma Lancet 2009 374 324 339 10.1016/S0140-6736(09)60221-X 19541364

[B21] Hanamura I Huang Y Zhan F Barlogie B Shaughnessy J Prognostic value of cyclin D2 mRNA expression in newly diagnosed multiple myeloma treated with high-dose chemotherapy and tandem autologous stem cell transplantations Leukemia 2006 20 1288 90 10.1038/sj.leu.2404253 16688228

[B22] Shi L Campbell G Jones WD Campagne F Wen Z Walker SJ Su Z Chu T-M Goodsaid FM Pusztai L Shaughnessy JD Oberthuer A Thomas RS Paules RS Fielden M Barlogie B Chen W Du P Fischer M Furlanello C Gallas BD Ge X Megherbi DB Symmans WF Wang MD Zhang J Bitter H Brors B Bushel PR Bylesjo M The MicroArray Quality Control (MAQC)-II study of common practices for the development and validation of microarray-based predictive models Nat Biotechnol 2010 28 827 38 10.1038/nbt.1665 20676074 PMC3315840

[B23] Popovici V Chen W Gallas BG Hatzis C Shi W Samuelson FW Nikolsky Y Tsyganova M Ishkin A Nikolskaya T Hess KR Valero V Booser D Delorenzi M Hortobagyi GN Shi L Symmans WF Pusztai L Effect of training-sample size and classification difficulty on the accuracy of genomic predictors Breast Cancer Res 2010 12 R5 10.1186/bcr2468 20064235 PMC2880423

[B24] Zou Y Liu J Wang S Zha L Xu Z CCIndex: A Complemental Clustering Index on Distributed Ordered Tables for Multi-dimensional Range9th IFIP Int Conf Netw Parallel Comput 2010 247 261

